# Towards Outlier Sensor Detection in Ambient Intelligent Platforms—A Low-Complexity Statistical Approach †

**DOI:** 10.3390/s20154217

**Published:** 2020-07-29

**Authors:** Diego Martín, Damaris Fuentes-Lorenzo, Borja Bordel, Ramón Alcarria

**Affiliations:** 1 ETSI de Telecomunicación, Universidad Politécnica de Madrid, Av. Complutense 30, 28040 Madrid, Spain; damaris.fuentes.lorenzo@upm.es; 2 ETSI Sistemas Informáticos, Universidad Politécnica de Madrid, Calle de Alan Turing s/n, 28031 Madrid, Spain; borja.bordel@upm.es; 3 ETSI en Topografía, Geodesia y Cartografía, Universidad Politécnica de Madrid, Camino de la Arboleda s/n, 28031 Madrid, Spain; ramon.alcarria@upm.es

**Keywords:** abnormal data, Ambient Intelligence platform, binary classifier, outlier detection, prediction model, sensor

## Abstract

Sensor networks in real-world environments, such as smart cities or ambient intelligent platforms, provide applications with large and heterogeneous sets of data streams. Outliers—observations that do not conform to an expected behavior—has then turned into a crucial task to establish and maintain secure and reliable databases in this kind of platforms. However, the procedures to obtain accurate models for erratic observations have to operate with low complexity in terms of storage and computational time, in order to attend the limited processing and storage capabilities of the sensor nodes in these environments. In this work, we analyze three binary classifiers based on three statistical prediction models—ARIMA (Auto-Regressive Integrated Moving Average), GAM (Generalized Additive Model), and LOESS (LOcal RegrESSion)—for outlier detection with low memory consumption and computational time rates. As a result, we provide (1) the best classifier and settings to detect outliers, based on the ARIMA model, and (2) two real-world classified datasets as ground truths for future research.

## 1. Introduction

In the two last decades, technology innovation has led to intelligent environments [1] such as smart homes [2], smart hospitals [3], or even smart cities [4]. IT (Information Technology) infrastructures such as sensors enable making decisions by providing real-time information of the environment to end users, leveraging interconnected devices in a huge number of domains. Sensor systems offer real-time monitoring information on a wide variety of contexts, such as health monitoring, environmental applications, transport utilities, or manufacturing [5]. Sensors can provide measurements about almost everything—temperature, health parameters, weather, material composition.

In sensors-based systems—Ambient Intelligence (AmI) platforms, smart-city applications, and so forth—the highly diverse and distributed data sources make the flow and interoperability among devices a complex challenge [6]. On a conceptual level, a main aspect to deal with is due to the different types of data to manage. The solution that emerged to manage this problem is the use of techniques such as semantic technologies [7], where data information from different sources is integrated into a common “semantic graph”, forming a shared vocabulary or ontology.

However, on a quality level, a key element in the use of multiple sensors in this kind of platforms is the reliability of the distributed information they are actually delivering. In large-scale platforms with so many devices, it is very common to observe sensors injecting corrupt information into the overall system, in most cases due to a malfunction of either the data transportation or the sensors themselves [8]. This causes the platform to deliver inaccurate information, losing end-users’ confidence, and forcing them to filter and clean the data, which may be an unfeasible task.

Monitoring erratic sensors can:

- Report about incorrect observations, allowing data managers to isolate and handle the point of failure where the corrupted data have been injected.
- Improve data reliability.
- Maintain the consistency and integrity of AmI databases.

**Figure 1:**
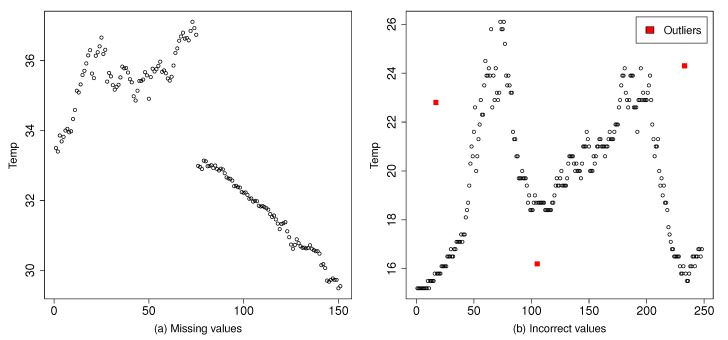
Examples of outliers: (**a**) from missing observations; and (**b**) from abnormal observations.

As Figure 1 illustrates, there are two types of incorrect or erroneous data measurements—(1) correct values after missing data—that is, when a sensor has failed or has been removed and no value is provided in the large-scale system until a time later; and (2) incorrect values, that is, when a sensor has failed in giving the correct measure, because of a sensor failure, an attack, and so forth, and an abnormal and inaccurate value is provided. In both cases, the erratic observation is considered as an *outlier*—out of the normal data pattern of the dataset.

Outlier detection finds extensive use in a wide variety of applications such as fraud detection for credit cards, health care anomalies, intrusion detection in computer networks, production faults in factories or military surveillance for enemy activities [9].

**Figure 2:**
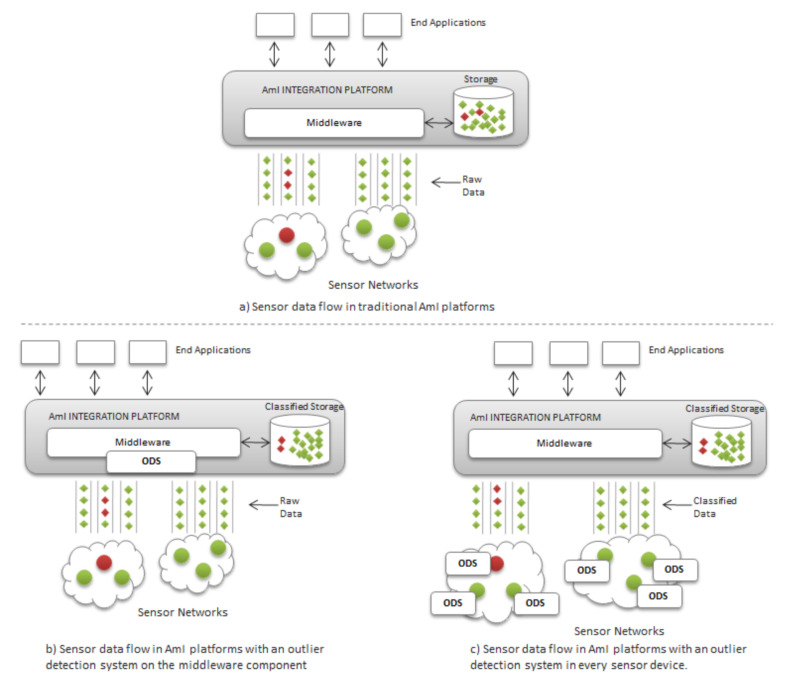
Possible outlier detection systems of erratic sensors in AmI platforms.

After working in our previous research with Fiesta-IoT [10], an AmI platform, we observed that sensors may eventually produce and deliver corrupted information into the overall platform. For this large-scale context, it is crucial to implement a high-accuracy outlier detection system whose requirements in terms of calculation time and storage are maintained to a minimum. As Figure 2 illustrates, an Outlier Detection System (ODS) may be set either into each separate sensor with limited computational power (Figure 2c), or into the integration AmI platform (Figure 2b) which, in the case of Fiesta-IoT, may tackle more than 100,000 sensor devices. These two ways can operate:

- *Online*: If the ODS is set into each device, it is suitable for analyzing streams of sensor observations, providing classifications with low latency.
- *Offline*: The ODS can also process a particular collection of observations offline to classify a complete history of data from a sensor.

In this research work, we examine three binary classifiers based on statistics to detect outliers produced and delivered by sensors in a large-scale platform with multiple devices. More specifically, our approach (a) collects real-world datasets as ground truths; and (b) analyzes the performance of those models in these datasets to detect both types of outliers. The three statistical predicting models we evaluate in the present work are ARIMA (Auto-Regressive Integrated Moving Average), GAM (Generalized Additive Model), and LOESS (LOcal RegrESSion), and they are oriented to particular time series. Time series are sets of numerical data observed with a fixed frequency—such as temperature or humidity—and are used in weather forecasting, econometric, mathematical finance, communications engineering, and so forth.

These specific models are selected due to their low computational time. The experimental analysis in this approach resumes a comparison among the different classifiers in terms of accuracy and other related metrics, and identifies the ARIMA-based classifier as the best technique for outlier detection.

This paper is structured as follows. Section 2 makes a general overview of the techniques already applied to outlier identification. Section 3 introduces our solution. Section 4 explains the experimental analysis of our approach, including dataset, models and parameters used, and ground truths applied for the evaluation. Section 5 shows and discusses the results obtained after the analysis. In the end, Section 6 exposes conclusions and final remarks.

## 2. Background and Related Work

The term *outlier* is referred to as any data object that does not comply with the general behavior or model in a data set. Outliers correspond to observations that deviate from other observations in a sample and do not conform to an expected pattern or other items in a dataset [9]. They may refer either to inconsistent data or good data that may point missing values. The existing works explained in the next subsections apply different approaches to detect outliers, which can be mainly grouped into (a) statistical techniques and (b) data-mining or machine-learning algorithms.

Both types can also be divided into unsupervised or supervised methods. Observations on unsupervised approaches are not previously labeled—previously classified as outliers or not. In supervised methods, samples of both normal and outlier values are used to construct models that identify new observations as one of the two classes.

The following subsections list some of the most relevant works according to the main kind of approaches.

### 2.1. Statistical Models

Essentially, statistical methods for anomaly detection are based on comparing the observed data with expected values. Liu et al. [11] propose LPS (Local Projection Score) to represent the degree of deviation of an observation relative to its neighbors. The nearest neighbors are first obtained for a given observation; then, the low-rank approximation, calculated from the nearest neighbors, is used to calculate the LPS. Observations with higher LPS were considered to be points with a high probability of being outliers. Here, the suitable LPS threshold was difficult to determine without information about anomalous observations, that is, with unsupervised datasets.

Markov models have also been used in Reference [12], which takes into account the last few commands (rather than the last single command) of a user in a UNIX system in order to determine the next normal command and avoid abnormal intrusions. However, the authors express their concerns about the large computing requirements needed to build the profiles.

Reunanen et al. [13] use LR (Logistic Regression) to predict outliers in streams of sensor data. The approach predicts the occurrence of outliers in *t* time steps in the future; however, it has to use labeled data—normal and anomalous observations—previously identified with an auto-encoder algorithm.

The work in Reference [14] uses an enhanced ARIMA algorithm for erratic traffic observations in wireless sensor networks. The ARIMA model is used in fitting time series data to make predictions. Their improved method has good accuracy and low complexity, but their model is updated after each time sliding window—the sliding window is the historical data used to predict the future data.

### 2.2. Machine-Learning Models

Most works based on machine-learning techniques apply unsupervised algorithms. These algorithms are usually distance-based methods, such as the work in Reference [15], where an outlier is defined as an observation that is *D* minimum distance away from a percentage *p* of observations in the dataset. The approach uses the KNN (K-Nearest Neighbor) algorithm; however, even though the authors state distance-based methods improve accuracy and efficiency, they do not provide quantitative results. The approach in Reference [16] uses a density-based anomaly detection called LOF (Local Outlier Factor), but a drawback of this technique is the $O(n^{2})$ complexity required.

Clustering-based algorithms are also one of the common approaches selected for anomalies detection. In Reference [17], an improved k-means clustering algorithm is proposed to the problem of outlier detection. The enhanced algorithm makes use of noise data filter and uses a density-based method. However, the drawback of this approach is that while dealing with large scale data sets, it takes more time to produce the results. In Reference [18], authors compare the k-means algorithm and the NOF (Neighborhood Outlier Factor) algorithm to identify intrusion detection in computer network traffic flows. The k-means approach groups the traffic flow data into normal and abnormal clusters. NOF algorithm [19] calculates an outlier score for each flow record, whose value decides whether a traffic flow is normal or abnormal. Overall, k-means approach is slightly more accurate and precise and has a better classification rate than NOF. Also, k-means precision increased as compared to NOF when the size of the dataset increased. In terms of performance, the outcome shows that the k-means algorithm consumes 10% to 20% of the CPU and takes approximately 5–10 s to execute; on the other hand, NOF consumes 50% to 60% of the CPU and takes approximately 40–50 s to execute on all the datasets.

As supervised approaches imply that both normal and anomalous observations are classified in the training dataset, and this collection may be difficult to obtain, the authors of References [20,21] propose hybrid semi-supervised anomaly detection models for high-dimensional datasets. In semi-supervised approaches, only normal samples are available in the training set; that is, the user cannot obtain information about anomalies. Unknown samples are classified as outliers when their behavior is far from that of the known normal samples. In Reference [20], they propose an anomaly detection model that consists of two components: a deep auto-encoder (DAE) and an ensemble KNN graphs-based anomaly detector, whose consuming time is a quadratic function, $O(n^{2})$. In Reference [21], their hybrid approach is based on k-means clustering and Sequential Minimal Optimization (SMO) classification, whose consuming time has a complexity of $O(n^{3})$.

Some works have paid attention to real large-scale environments with sensors. The work in Reference [22] proposes a classification approach for outlier detection in wireless sensor networks (WSNs), where dynamic nature and resource limitations have to be considered. This approach tries to identify outliers with high detection accuracy while maintaining the resource consumption of the network to a minimum, comparing random tree and random forest algorithms. Random tree offered better results, with an accuracy of 89% and a false positive rate of 11%. However, the time complexity is $O(n^{2})$ in its classification phase. In Reference [23], authors use real data from a smart city and compare frequently used anomaly detection techniques. As a result, they conclude that one-class Support Vector Machines is the most appropriate technique, with a true positive rate at least 56% higher than the rates achieved with the other compared techniques in a scenario with a maximum false positive rate of 5% and a 26% higher in a scenario with a false positive rate of 15%.

In the last years, deep learning-based anomaly detection algorithms have become increasingly popular, as reviewed in Reference [24], but there are concerns about the computational complexity of these techniques in real application domains.

## 3. Proposed Solution

As shown in the state of the art, most of the works for anomaly detection apply machine-learning approaches with considerable time complexity. Some of them require also storage resources that may be limited in large-scale, heterogeneous environments, which necessarily imply new light-weight approaches that can involve as less computation and storage resources as possible, identifying inappropriate data very rapidly.

AmI environments integrate vertical sectors, such as healthcare, cybersecurity, or environmental data, in order to improve the quality of human life [10], so it is crucial to explore efficient procedures to detect abnormal data. Therefore, the main goal of our approach is to find an appropriate method to detect outliers out of stream data sensors or in the joint distribution of sensor readings, in large-scale AmI platforms, where the resource consumption of the environment must be set to a minimum. The requirements of the method are the following:

- The method must be suitable for large datasets and must require a small amount of memory.
- The method does not have to require training data or semi-supervised techniques, which increment the computational time, and should have O(n) complexity.
- The method must provide results with a high value of accuracy.

Before the analysis of any particular model, it is important to define the streaming data source. Some existing works apply datasets with normal samples, and then add artificial values for abnormal data; this procedure, though, may not reflect the distribution of real outliers [9,25]. The specific context of this research—sensors in real world—requires a benchmark with a reliable, realistic, and sufficiently large quantity of data. Due to its characteristics, the analysis proposed in this work is executed with values taken from the “Sofia Air Quality Dataset” [26], a huge data collection with outdoor-sensors observations located in the city of Sofia, Bulgaria. This dataset represents observations taken every 150 s by 168 environmental sensors of pressure, temperature, and humidity during the month of July 2017. More specifically, the time series selected come from temperature measurements.

The research questions that drive this work are the following:

- Research question 1 (**RQ1**): Outliers due to missing values can be detected with high accuracy, low storage, and computational complexity?
- Research question 2 (**RQ2**): Outliers due to incorrect values can be detected with high accuracy, low storage, and computational complexity?

After the study of existing previous approaches—Section 2—and to satisfy the need of a model of low time complexity, this work analyses three prediction models, ARIMA, GAM, and LOESS to evaluate the best binary classifier in terms of accuracy, time, and resource consuming.

These algorithms are commonly applied to time series forecasting and require less time and storage requirements than machine-learning techniques [27]. There are more options for time series forecasting, such as Spline, Bayesian structural model, linear regression modeling, Holt-Winters exponential smoothing modeling, dynamic harmonic regression, long short-term memory, Kallman filters, and so forth. However, we have adopted ARIMA, GAM, and LOESS because of their widespread use and the relevant results offered in research of time series prediction [28,29,30,31,32].

## 4. Experimental Analysis

**Figure 3:**
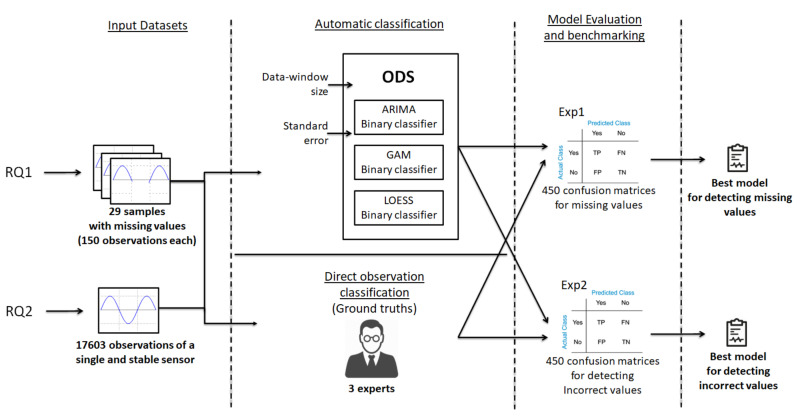
Experimental analysis methodology.

Figure 3 describes the overall process followed in our approach. This methodology (ODS, Outlier Detection System) addresses the two research questions above—RQ1) if outliers from missing observations can be detected; and RQ2) if outliers from abnormal observations can be detected. The solutions of these two inquiries are explored in two experiments respectively, Exp1 and Exp2, and they must accomplish the resources constraints of large-scale environments. For this commitment, we work with three prediction models (ARIMA, GAM, and LOESS), fitting a set of main parameters (data window size and allowed standard error) to find the best combination to maximize the result.

For an accurate and realistic evaluation, we have developed two ground truths from the whole Sofia Air Quality Dataset in order to compare the results of the classifiers to this real set—a ground truth for RQ1 with samples including missing values; and a ground truth for RQ2 with samples including incorrect observations.

The next subsections explained thoroughly all these elements in our analysis.

### 4.1. RQ1 and RQ2 Datasets

The first dataset used in Exp1 to satisfy RQ1 is composed of a set of 29 samples extracted from the data source that meets a specific characteristic—there are missing values among the observations. Each of these samples contains 150 observations taken in chronological order, which results in a benchmark collection of 4350 observations.

**Figure 4:**
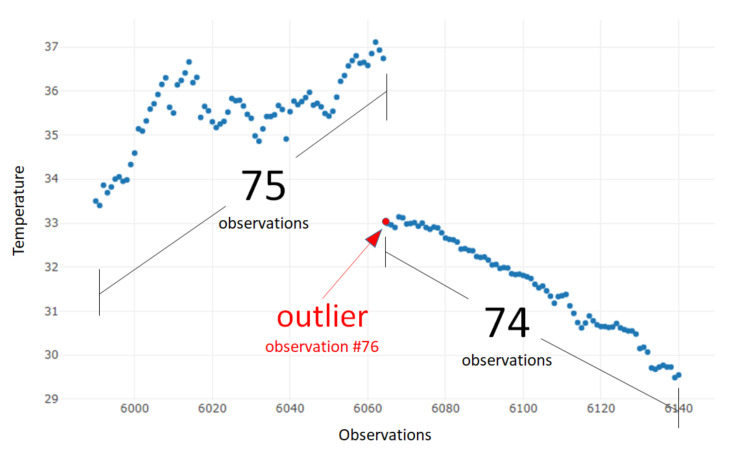
Example of a single sample vector with missing values.

We performed a semi-automatic search among all the sensor observations from the original data source. As temperature measurement is a type of time series—every observation contains a time element—it is clear when the sensor has stopped delivering measurements. First, an automatic selection took out data fragments with missing data. Then, the most representative fragments were chosen manually; due to the stability of the sensor observations, we were able to find those samples with the same pattern (illustrated in Figure 4). In every sample vector, the 76th value has a clear time lapse from the previous 75th value; the rest of the following observations do not have any significant time difference.

**Figure 5:**
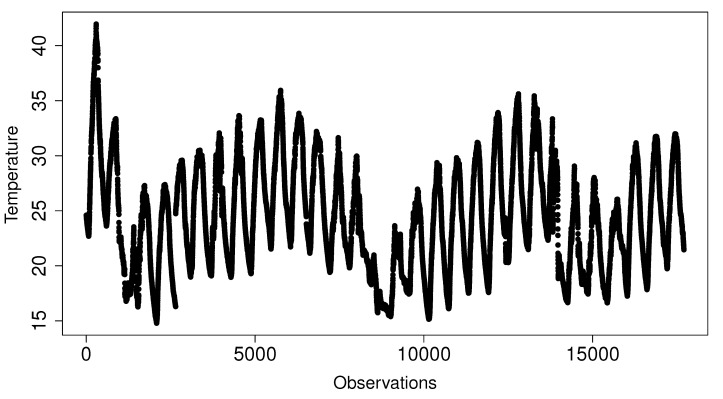
Observations taken by Sensor-1850, Sofia Air Quality Dataset [26].

The second dataset, elaborated for Exp2 to analyze RQ2, is formed by every observation of one specific temperature sensor—sensor 1850—from the original data source. This sensor measured stable observations every 150 s during a period of a month, July 2017. The number of total observations in this set is 17,603 values. Figure 5 shows the data stability in these observations.

### 4.2. RQ1 and RQ2 Ground Truths

In order to rank and evaluate the classifiers, we have created two ground truths for this research.

The first ground truth is originated from the first dataset explained, composed of 29 samples, each one of 150 observations. Each observation has been semi-automatically classified as normal or outlier—an outlier here is easily classified because of the time-lapse reflected. This ground truth is used in Exp1 to evaluate the performance of the classifiers for RQ1.

The second ground truth is formed out of the second dataset; it is composed of the complete manual classification of the 17,603 values in that benchmark and it serves to Exp2. The direct validation has been made by three experts who participated in the experiment.

These three experts were students in their final year of the master’s degree “Master of Science in Telematic Services and Networking Engineering”, where they attended several subjects on IoT, signals, and sensoring. Before starting the manual classification, the experts received special training on what is considered a correct or incorrect value.

In order to carry out this arduous task, we used very simple software to present the data to be classified by means of samples of 200 observations. Using this software, the experts labeled each value as either *normal* or *outlier* according to the instructions they received in the training phase. As a result, each value is tagged with three assessments (one per expert), which are finally unified: a particular observation is considered as an outlier if it was labeled as outlier by two or more experts. Both ground truths are published together with this article in the journal site (in Supplementary Materials).

### 4.3. Binary Classifiers and Parameters

In this research work, we compare three binary classifiers based on three statistical inference techniques—ARIMA, GAM, and LOESS. The three classifiers make use of the ability to predict the next values of these inference models to classify a single value ahead as an *outlier*—if it is far from the predicted value—or *normal* otherwise.

The ARIMA model uses observations from previous time steps to predict the value for the next time step, using a particular regression equation [33]. ARIMA (*p*, *d*, *q*) is the standard setting parameters used [34]: *p* refers to the number of lag observations included in the model to adjust the line that is being fitted; *d* indicates the number of differencing transformations needed by the time series to get stationary, and *q* refers to the size of the moving average window.

LOESS is a non-parametric local regression method where a smooth function may be properly fitted by a low degree polynomial in a particular close subset of any point of a dataset by a moving window strategy [35]. In the smoothing process, the sample of a timer series is adjusted so that individual values that are higher than the immediately adjacent values are moderated, and the same with lower ones. In the weighted polynomial regression used, more score is given to values near the target observation, and less score is given to values further away.

GAM is not restricted by the assumption in regression that requires predictor and outcome variables to move in a straight line; it is based on non-linear smooth functions instead of individual prediction variables [36]; that is, replaces a linear regressive function by a sum—additive—of smooth functions.

For the models’ internal settings, the best numbers for ARIMA standard parameters are selected according to the Akaike Information Criterion (AIC) [37]. In the case of the binary classifiers where the GAM and LOESS models are used, there is no need to adjust any additional internal parameters.

**Figure 6:**
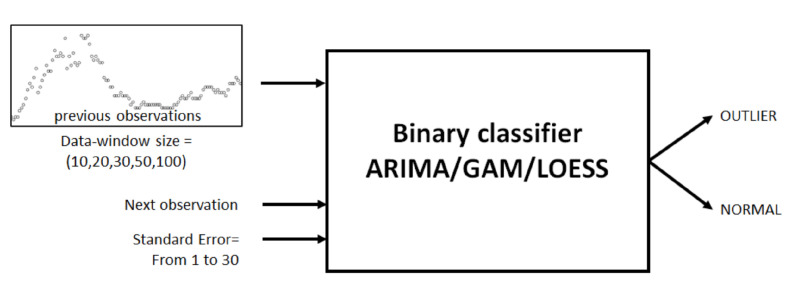
Black-box diagram of a binary classifier.

The black-box diagram in Figure 6 illustrates the binary classifiers proposed in this research and the two parameters needed to classify the next observation.

The three binary classifiers are tested with the following parameters and values:

- *Data-window size*—The classifiers are feed with previous observation values in order to perform the predictions; this collection of values is named as *data window* and its size is called *data-window size*. Values considered are 10, 20, 30, 50, and 100.
- *Standard error*—Any prediction model, in addition to the predicted value, provides a standard error on that prediction, which is the standard deviation of its sampling distribution. The three binary classifiers use this value to determine whether the next observation analyzed is an outlier. The classifiers will accept an allowed *standard error*, above or below the predicted value, to consider a particular observation as *outlier* or *normal*. Values considered range from 1 to 30.

The challenge is finding the appropriate parameters’ combination—data-window size and standard error—to both accurately identify true outliers—true positives—and avoid the classification of normal observations as outliers—false positives.

**Figure 7:**
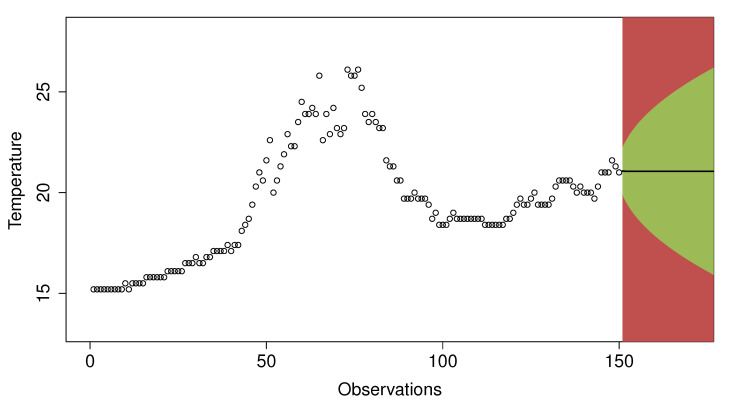
Predictions made for the 150th value. The green and red areas cover the zones where the next observation would be considered as normal or outlier respectively.

Figure 7 illustrates an example of a prediction made for the 150th observation. The green-colored area represents the observations that would be considered as normal, while the red area would correspond to outliers. In the example shown in the figure, an observation is considered as an outlier if it exceeds twice the standard error of the prediction. If the next value is considered as normal, it will be added to the data window to make the next predictions; otherwise, it will not be added. This way, the values considered as outliers do not interfere in future predictions.

**Figure 8:**
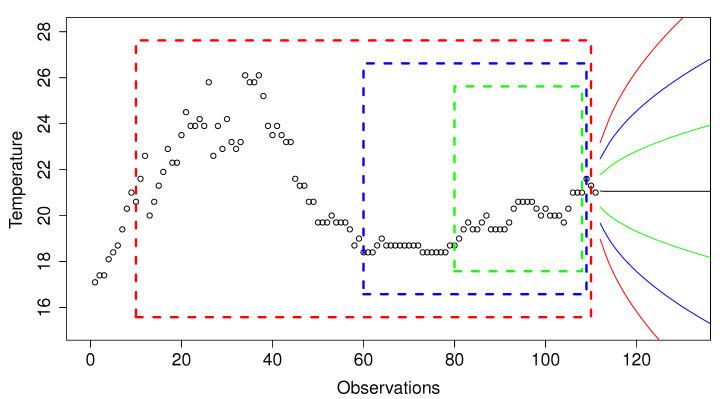
Example of data-window sizes and the standard errors allowed for classifying the next observation. Dotted red, blue, and green lines represent a data-window size of 100, 50, and 30 values respectively. The solid red, blue, and green lines mark 3, 2, and 1 times the standard error respectively.

Figure 8 shows three data windows in blue, green, and red boxes. The green window uses 10 observations to predict the next one, the blue window uses 20 values, and the red window uses 30 values. The area bounded by green lines shows the predictions made by a model using a standard error of 1, and the areas bounded by blue and red lines indicate a standard error of 2 and 3, respectively.

**Figure 9:**
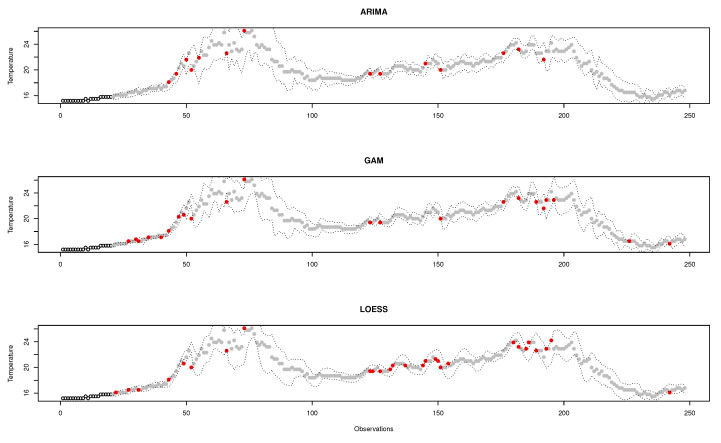
Example of classification made by the three proposed binary classifiers.

Figure 9 shows an example of a classification made by the three binary classifiers. All of them use a data window of 20 observations to make the predictions and a deviation of 2 for the standard error. The area limited by the dotted lines accommodates normal observations; outside that area, observations are considered outliers (shown as red dots). Each classifier identifies a different set of observations as outliers; although there is often consensus, the same assessments are rarely achieved. It is therefore important to find the perfect combination that better responds to the research questions proposed in this work.

## 5. Results and Discussion

The research questions RQ1 and RQ2 guide the experimental analysis accomplished in this research. The next subsections explain the first experiment, Exp1, conducted to solve RQ1, and the second experiment, Exp2, to solve RQ2. A discussion of the overall analysis is presented at the end.

The classifiers are tested with 450 combinations. After the testing phase, the resulting classifications of Exp1 and Exp 2 are compared to RQ1 and RQ2 ground truths respectively, generating 450 confusion matrices. The main performance indices obtained are accuracy, recall, precision, $F_{\beta }$-Score, true positives (TP), true negatives (TN), false negatives (FN) and false positives (FP).

The rankings are ordered using the $F_{\beta }$-Score, which measures the accuracy regarding the precision and the recall; in fact, it is defined as the harmonic mean of the precision and recall, as shown in Equation (1).

(1) $$F_{\beta }\mathrm{-Score}=(1+\beta^{2})\cdot \frac{precision\cdot recall}{(\beta^{2}\cdot precision)+recall,}$$

where:

- precision = true positives/(true positives + false positives)
- recall = true positives/(true positives + false negatives).

In addition, the $F_{\beta }$-Score allows to give more weight to recall with respect to accuracy by customizing the value of β, see Equation (1). This is important for the experiments because normal observations in the datasets are much more predominant than the outlier values. The dataset for Exp1 contains 29 outliers against 4321 normal values; the dataset for Exp2 contains 534 outliers and 17,069 normal values. In an AmI context, we prefer to give more importance to the detection of outliers (true positives), even though normal observations could be classified as outliers (false positives).

Equation (2) shows the $F_{\beta }$-Score expressed in terms of true positives, false negatives and false positives metrics.

(2) $$f_{\beta }\mathrm{-Score}=\frac{(1+\beta^{2})\cdot true\;positive}{(1+\beta^{2})\cdot true\;positive\;+\;\beta^{2}\cdot false\;negative\;+\;false\;positive.}$$

### 5.1. Results for RQ1

Table 1 shows a summary of the first four positions in the classification ranking. It is ordered by the $F_{1}$-Score, although it does not have much relevance since there are 20 results which compare equal; they perfectly classify all the observations.

However, there are combinations that launch false predictions, as shown in Table 2.

**Figure 10:**
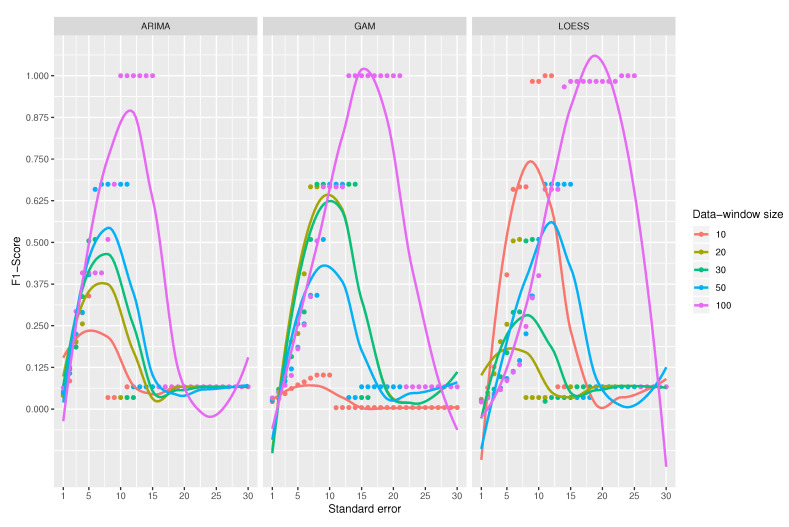
Comparison chart among functions, data-window sizes, and standard errors. Exp1.

**Figure 11:**
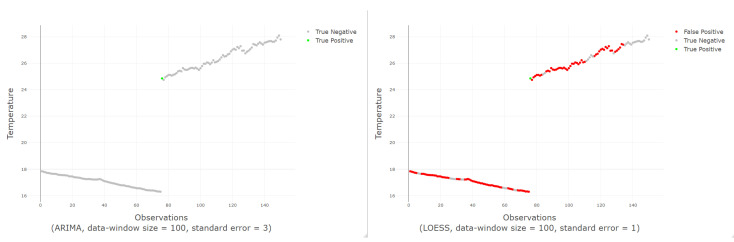
On the left, an example of a good classification; on the right, an example of a bad classification.

A comparison among the $F_{1}$-Score of the classifiers is shown in Figure 10. At first sight, LOESS predictive model obtains the best results for $F_{1}$-score compared to ARIMA and GAM models. Among ARIMA and GAM models, GAM works well except for the combination that uses 10 observations for the data-window size, which is clearly the worst. Figure 11 shows an example of a good and bad classification with the ARIMA and LOESS models respectively.

A data-window size of 100 observations yields the best scores for all classifiers. For the ARIMA-based classifier, the scores are perfectly ordered by data-window size; the larger the data-window size, the better the classification. In the case of the classifier using GAM, the data-window size of 10 observations offers the worst results, but for the other sizes, it does not seem to be a relation. When using LOESS, chaotic results appear; best results are for a window of 100 observations (the largest) and 10 observations (the smallest). That is why the use of LOESS is not recommended even if it gets sometimes the best results.

For the case of ARIMA’s predictive model, the maximum values are obtained when the standard error ranges from 10 to 15. In the case of GAM, it has a good performance for all combinations where the standard error is between 13 and 23; however, when using a data-window size of 10 observations, it gets a very low score. In the LOESS model, the performance gets better with a standard error between 15 and 25; its best data-window size is 100.

The three binary classifiers perform the best when the data-window size is higher, obtaining the optimum value with a data-windows size of 100 observations. It is not considered to further increase the window size because this would mean having more data in memory and increase the complexity of space, an aspect that goes against the research objectives.

Although LOESS apparently achieves better results, its use is discouraged as the results for the different data-window size values do not seem to make sense and so it is considered unpredictable. On the contrary, the recommended model is ARIMA, which yields predictable results for different data-window sizes. As a final conclusion, the best classifier to detect missing values is that using an ARIMA prediction model, with a data-window size of 100 observations and a standard error between 10 and 15. The value of the standard error should not be increased too much since missing values will be left undetected.

### 5.2. Results for RQ2

To respond to RQ2, Exp2 uses the input data set described in Section 4.1. The labeling of the classifiers is compared with the ground truth described in Section 4.2 to check their performances.

Table 3 shows a summary of the ranking obtained. The table is ordered by the $F_{3}$-Score. This ranking uses a β = 3 in order to apply more weight to recall; in doing so, outlier detection gives more importance to identifying outliers than to predict false positives.

The best score is achieved by the ARIMA-based prediction classifier, with a data-window size of 30 and a standard error of 2. This combination also gets the first place if the ranking is ordered by accuracy, recall, or precision. As in Exp1, the ARIMA prediction function can be used effectively to determine incorrect observations, but it must be applied to a particular combination of parameters; identifying this type of outliers can be harsher—in terms of settings—than identifying missing values.

**Figure 12:**
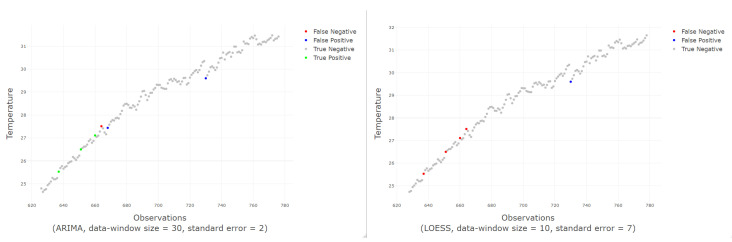
On the left, an example of good classification; on the right, an example of bad classification.

Figure 12 shows an example of good and bad classifications made by the ARIMA and LOESS model respectively.

**Figure 13:**
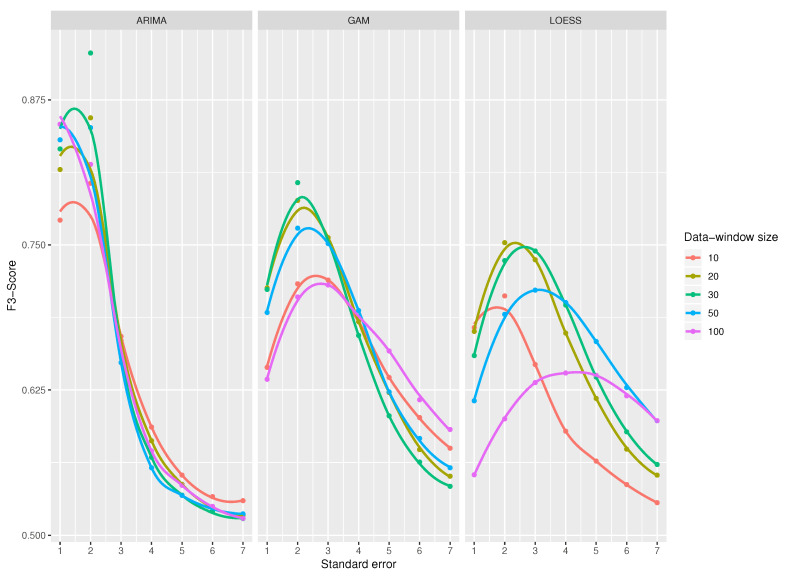
Comparison chart among functions, data-window sizes and standard errors. Exp2.

A comparison of all classifiers is shown in Figure 13 for a standard error from 1 to 7 since from value 7 the results are very close to 0. The results from 8 to 30 were omitted in the figure, as they do not provide any improvement in the results. All the functions obtain their maximum value when the standard error has a value around 2 and from a value of 4, the results start to decay. It is also noticeable that ARIMA-based classifier gets better results compared to GAM and LOESS-based classifiers. Among GAM and LOESS predictive models, GAM model gets better scores than LOESS model.

There are two concerns to note. First, as function curves get flatter, the standard error has less impact on the resulting metrics. The ARIMA-based classifier has a more slope curve, a peculiarity that affects the metrics more than the standard error. Second, the data-windows size seems not to affect the ARIMA-based classifier; however, this parameter has a great impact in the $F_{3}$-Score of GAM and LOESS-based classifiers.

When using an ARIMA model and a standard error of 2, the best result is obtained for a data window of 30 observations. For standard errors higher than 2, the function starts to decay drastically and there is hardly any difference among the data-window sizes.

The classifier that uses GAM as a prediction model, and for a standard error equal to 2, achieves the maximum values for every data-window sizes; that is, the data-window selected does not affect the results—the functions are very close together in Figure 13. It is interesting to note that the curves in the extreme values for the data windows (100 and 10 observations) obtain very low results for $F_{3}$-Score; the best score is obtained with a data-window size of 30, as in the case of ARIMA.

The curves obtained by LOESS model are flatter compared to those of GAM and ARIMA, meaning that the standard error does not affect the score as much; like the previous ones, the maximum values are achieved when the standard error has a value between 2 and 3, and again, the data-window sizes with best results are 20 and 30.

For the three prediction models, the variable that most affects the detection of outliers is the standard error, while the data-window size does not seem to affect so much. Then, the best combination to use to answer RQ2 is a data-window size of 30 (or close), a standard error of 2, and, whenever possible, a predictive model based on ARIMA.

#### 5.2.1. Analysis of False Positive Rate (FPR)

The FPR metric is also known as *fall*-*out* or *false alarm* ratio. In our approach, this rate represents the normal observations classified as outliers; therefore, the lower, the better. Even though the false positives are higher than the true positives detected by the three classifiers, this ratio is very low; Table 4 shows the best classifier has a FPR under 0.06, which is a very significant result. In our approach, the best results also yield mislabeling with normal instances; that is, they are classified as outliers. However, it is preferred that classifiers make these types of errors as long as they are good at classifying outliers as such; the metric $F_{3}$-Score gives more weight to a good classification of outliers. In spite of this, general results are promising, since the FPRs reflect very low values because there are many normal observations (17,069) that are classified correctly in this environment.

#### 5.2.2. Analysis of False Negative Rate (FNR)

The FNR metric measures the ratio of observations that are positive—outliers—but were erroneously classified as negative—normal; as in FPR, the lower this rate, the better. The best classifiers obtain a rather low FNR, having values below 0.25%, which is significant. The best classifier only failed 61 times, classifying outliers from a total of 17,603 observations. This is a very good result, and it confirms that our approach is a suitable solution for outlier detection.

#### 5.2.3. Analysis of True Negative Rate (TNR or Specificity)

The TRN measures the ratio of actual negative observations that are correctly classified as such; that is, it is the percentage of normal observations identified as normal—that is, not incorrectly classified as outlier. In this rate, the higher the value, the better. Table 4 shows that the best classifiers achieve very high rates for Specificity, above 90% in most cases, demonstrating that they are able to discern whether a value is normal or outlier. These good rates are achieved because, in our context, there is a very high ratio of normal observations (17,069) compared to outlier observations (534), and so the classifiers perform well.

#### 5.2.4. Analysis of True Positive Rate (TPR, Recall, or Sensitivity)

The TPR metric is the ratio of detection of true positives. It measures the ratio of actual outliers that are correctly identified as such; it can be expressed as a percentage of the outliers that were correctly identified as outliers. It is a very important metric used to measure the performance of classifiers since it takes into account true positives as well as false negatives. The results for the recall shown in Table 4 are quite high values for the top-ranked classifiers.

### 5.3. Discussion

As seen in the previous sections, the approach proposed in this paper, based on the application of predictive models, is an effective, realistic, and low-cost computational solution to detect outliers from both missing values (RQ1) and incorrect observations (RQ2). The model that achieves the best scores for RQ1 and RQ2 in terms of accuracy is ARIMA, but it is very important to note that the model settings must be different depending on the specific goal—the parameterization for RQ1 is not valid for RQ2 and vice versa. Thus, for a real-world implementation that wants to address the two research questions proposed in this paper, and as a suggestion to reduce the computational cost, it is proposed to use the ARIMA model working in two different sets of instances, each of them with the appropriate configuration suggested in the previous sections.

## 6. Conclusions

Outlier detection has gained importance in the last two decades with the use of real-world applications. Domains where anomaly detection is crucial include financial fraud detection, computer network intrusion, data quality control, health supervision, and so forth. There exist a lot of work in the area of machine-learning models; however, large-scale platforms such as AmI environments or smart cities require algorithms that meet computational time and memory constraints. AmI platforms can be augmented constantly with sensor devices with high constraints—computing power, communication, and storage limitations; thus, we aim to resolve a scalable solution for real-world platforms with such limited and distributed nature.

This paper studies three statistical prediction models—ARIMA, GAM, and LOESS—for the identification of outliers, both from missing and incorrect values. The two main advantages of these classifiers are (1) their low computational complexity, O(n); and (2) the memory consumption needed for their functioning, which is extremely low in our experiments—the data-window size does not exceed 100 values.

The use of the classifiers based on the predictive models presented in this research work is a feasible solution for the detection of missing values and abnormal observations in sensor data streams. The best classifier analyzed to detect outliers is based on the ARIMA prediction model. However, is extremely important to establish the correct values for its parameters. To detect missing values, the ideal settings are a data-window size of 100 observations and a standard error between 4 and 5. To detect incorrect or abnormal values, the ideal settings are a data-window size of 30 observations and a standard error of 2. This is the most crucial task in this research: finding the appropriate parameters for the two types of outliers.

Although temperature sensors were used in the experiment, this approach can be used to detect outliers in other types of sensors measuring a physical quantity. In the case of trying to classify data streams from another type of sensor, a previous analysis should be carried out in order to find the ideal settings that make binary classifiers work optimally.

Our study is significant in providing a) an in-depth understanding of the performance of the three statistical models analyzed for outlier detection; and b) two datasets as ground truths, whose observations are manually labeled by experts as either normal or outlier.

The global context of the sensors in a large-scale environment may be used for future research. The input of the models studied in this work is local, as one sensor data is used at a time to locate outliers in that particular sensor. Observations in the same time lapse from other different sensors may improve detection rates in one particular sensor device.

Other statistic and machine-learning algorithms with O(n) complexity may be proved, fitted, and evaluated to take advantage of their good accuracy rates, but minimizing their storage needs to turn them into a minimum.

Finally, further analysis may assess to prove if the three classifiers in our approach can be applied to other kinds of observations (e.g., humidity, precipitation), other nature (e.g., textual data), or with different measurement times. We plan to create an outlier detection assistant to, given a measurement type and the time lapse between the observations, the assistant resolves which model and settings fit better for the detection task. For this, the elaboration of ground truths from real data is crucial; as it is a very hard and complex task, it will surely be the subject of future research.

## Figures and Tables

**Table 1:** Ranking with the first four positions of the results in Exp1, ordered by $F_{1}$-Score.

Prediction Model	Data-Window Size	Standard Error	Accuracy	Recall	Specificity	$F_{1}$- Score	TP	TN	FN	FP
ARIMA	100	3	1.0	1.0	1.0	1.0	29	4310	0	0
ARIMA	100	4	1.0	1.0	1.0	1.0	29	4310	0	0
ARIMA	20	5	1.0	1.0	1.0	1.0	29	4310	0	0
ARIMA	10	6	1.0	1.0	1.0	1.0	29	4310	0	0

**Table 2:** Ranking with the last four positions of the results in Exp1, ordered by $F_{1}$-Score.

Prediction Model	Data-Window Size	Standard Error	Accuracy	Recall	Specificity	$F_{1}$- Score	TP	TN	FN	FP
GAM	10	14	0.8961	0.0345	0.9019	0.0044	1	3897	28	424
GAM	10	12	0.8961	0.0345	0.9019	0.0044	1	3897	28	424
GAM	10	13	0.8961	0.0345	0.9019	0.0044	1	3897	28	424
GAM	10	11	0.8832	0.0345	0.8889	0.0039	1	3841	28	480

**Table 3:** Ranking with the first 4 positions of the results in Exp2, ordered by $F_{3}$-Score.

Prediction Model	Data-Window Size	Standard Error	Accuracy	F_3_ Score	TP	TN	FN	FP
ARIMA	30	2	0.9431	0.9153	473	16,129	61	940
ARIMA	20	2	0.9281	0.8595	420	15,917	114	1152
ARIMA	100	1	0.7398	0.854	521	12,501	13	4568
ARIMA	50	2	0.9398	0.851	404	16,139	130	930

**Table 4:** False Positive Rate, False Negative Rate, Specificity, and Recall metrics for Exp 2.

Prediction Model	Window Size	Standard Error	FPR	FNR	Specificity	Recall	TP	TN	FN	FP
ARIMA	30	2	0.0551	0.1142	0.9449	0.8858	473	16,129	61	940
ARIMA	20	2	0.0675	0.2135	0.9325	0.7865	420	15,917	114	1152
ARIMA	100	1	0.2676	0.0243	0.7324	0.9757	521	12,501	13	4568
ARIMA	50	2	0.0545	0.2434	0.9455	0.7566	404	16,139	130	930

## Supplementary Materials

The Ground Truths are available online at https://www.mdpi.com/1424-8220/20/15/4217/s1.
